# Fluidity and phase transitions of water in hydrophobic and hydrophilic nanotubes

**DOI:** 10.1038/s41598-019-42101-4

**Published:** 2019-04-05

**Authors:** Mohamed Shaat, Yongmei Zheng

**Affiliations:** 1 Department of Mechanical Engineering, Zagazig University, Zagazig, 44511 Egypt; 2 Mechanical Engineering Department, Abu Dhabi University, Al Ain, P.O.BOX 1790 United Arab Emirates; 3 Engineering and Manufacturing Technologies Department, DACC, New Mexico State University, Las Cruces, NM 88003 USA; 4 Key Laboratory of Bio-Inspired Smart Interfacial Science and Technology of Ministry of Education School of Chemistry, and Beijing Advanced Innovation Center for Biomedical Engineering Beihang University (BUAA), Beijing, 100191 P. R. China

## Abstract

We put water flow under scrutiny to report radial distributions of water viscosity within hydrophobic and hydrophilic nanotubes as functions of the water-nanotube interactions (${{\epsilon }}_{sf}$), surface wettability (*θ*), and nanotube size (*R*) using a proposed hybrid continuum-molecular mechanics. Based on the computed viscosity data, ${{\rm{\epsilon }}}_{{\rm{sf}}}/{\rm{\theta }}-{\rm{R}}$ phase diagram of the phase transitions of confined water in nanotubes is developed. It is revealed that water exhibits different multiphase structures, and the formation of one of these structures depends on ${{\rm{\epsilon }}}_{{\rm{sf}}},\,{\rm{\theta }},$ R parameters. A drag of water flow at the first water layer is revealed, which is conjugate to sharp increase in the viscosity and formation of an ice phase under severe confinement (R ≤ 3.5 nm) and strong water-nanotube interaction conditions. A vapor/vapor-liquid phase is observed at hydrophobic and hydrophilic interfaces. A state of confinement is revealed at which water exhibits different multiphase structures under the same flow rate. The derived viscosity functions are used to accurately determine factors of flow enhancement/inhibition of confined water.

## Introduction

The study of the characteristics of nanoconfined water is important to expand our current understanding of advanced nanofluidics and give us the ability to engineer advanced nanoscale systems^1–4^. When water is confined within a nanotube or a nanochannel, it exhibits drastically different characteristics than bulk water^5–15^. Water in such nanoscale conveyors is affected by the wettability of the confining surface, water-surface interactions, and confinement size^7,16^. For instance, enhanced flow rates were determined when water flowed adjacent to hydrophobic surfaces^7,16–19^ and hydrophilic surfaces^17,20–22^. Water particles may stick to or leak through a superhydrophilic surface^15^.

The structure of water in nanotubes depends on the radial distribution of water-surface interactions. These interactions define the degree of the hydrophobicity/hydrophilicity of the confining nanotube and its ability to enhance/inhibit the flow and alter the structure of water. At the interface, a water depletion layer is usually formed due to the repulsive role of water-surface interactions^7,15,17,18,23–27^. This layer is distinguished with an intensive decrease in the water density. Beyond the depletion layer and at the first water layer, the density of water sharply increases and follows a radial distribution towards the nanotube center^7,12,15,17^. The observed structures of water in nanotubes revealed multiple phases of water at hydrophobic and hydrophilic interfaces^28–31^. These multiphase structures of water were revealed based on water density variations in the nanotube. However, the interpretation of the multiphase structures and phase transitions of water based on the viscosity variations would explore new phenomena of advanced nanofluidics. Therefore, reports on the variations of water viscosity between the confining surfaces are needed. Because of the water-surface interactions and the wettability of the confining surface, the viscosity (*which is a measure of the fluidity and a representation of the continuity of molecular interactions of the continuum*) varies as a function of the separation from the confining surface. The determinations of the shear force at different heights of a water-on-surface indicated a water viscosity variation with the separation distance^32,33^. In addition, the viscosity can be related to the density^34–38^, which would give a water viscosity profile if intersected with the water density profile. A great deal of probing the viscosity of water interfaces with superhydrophilic flat surfaces by means of atomic force microscopy has been done, which revealed the viscosity of water is 2 to 6 orders-of-magnitude greater than that of bulk water^17,33,39^. However, the experimental assessment of water viscosity inside nanotubes is a challenge.

Here, we present a simple but effective approach for identifying the viscosity of water confined in nanotubes using a hybrid continuum-molecular mechanics (HCMM) (*see Methods*). The radial distributions of water viscosity inside different hydrophobic and hydrophilic nanotubes are reported. First, we demonstrate that a velocity profile of water in a nanotube is non-parabolic, and hence its viscosity has a radial distribution. At the interface with the nanotube, the viscosity may be lower/higher than the bulk water viscosity depending on the water-surface interactions, surface wettability, and nanotube’s size. In addition, a drag in the water flow at the first water layer is revealed. This drag corresponds to a sharp increase in the water viscosity at this layer. Second, we show the phase transitions of confined water in hydrophobic and hydrophilic nanotubes. We show the formation of a multiphase structure when water is severely confined. A solid phase of water (ice) with viscosity multiple times higher than the one of bulk water is observed at the first water layer. At the interface, water vapor may form. Finally, we show that the accurate description of water flow in nanotubes is achievable only via the radial variation of viscosity. Thus, the factors of the flow enhancement/inhibition are accurately reported for different hydrophobic and hydrophilic nanotubes.

## Results

Using the developed HCMM (*see Methods*), the radial variations of water viscosity inside hydrophobic and hydrophilic nanotubes were determined. 540 simulations of water flow in hydrophobic and hydrophilic nanotubes with different radii (R), water-surface interaction energy (${{\epsilon }}_{sf}$), and wettability (*θ*) were carried out. The Lennard-Jones (*LJ*) parameters of water-water interaction were defined by ${{\epsilon }}_{ff}=0.6502\,{\rm{kJ}}/{\rm{mol}}$ and *σ*_*ff*_ = 0.3169 nm. To study the influence of the water-surface interactions, different *LJ* potentials were introduced between water particles and the nanotube. The viscosity profiles, velocity profiles, and flow rates of the 540 cases were obtained. To show the accuracy of the proposed HCMM, comparisons with over 90 cases of the experimental and molecular dynamics (MD) results in the literature were carried out (*see Supplementary Information – Model Validation*).

### Interplay between Drag of Water Flow and its Viscosity

**Figure 1:**
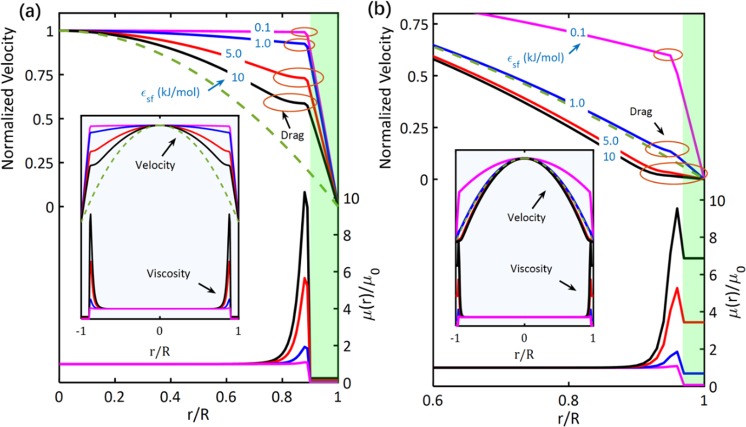
Drag of water flow in nanotubes. Velocity and viscosity profiles of water flow through (**a**) 5 nm-nanotubes and (**b**) 12 nm-nanotubes with different water-surface interactions (${{\epsilon }}_{sf}$). The drags of the water flow are located by brown circles. The interface between water and nanotube is green highlighted. Insets show the complete velocity and viscosity profiles. Velocity profiles are normalized, *i.e. v*(*r*)/*v*_0_ where *v*_0_ is the velocity at the nanotube center (*r* = 0). All the simulations were carried out with *σ*_*sf*_ = 0.5 *nm*. The green-broken curve is the velocity profile of the no-slip Hagen–Poiseuille flow.

Velocity profiles and radial viscosity distributions of water flow in nanotubes with diameters 2 R = 5 nm and 2 R = 12 nm (*i.e*. R is the nanotube’s radius) are depicted in Fig. 1 for different values of the water-surface interaction energy (${{\epsilon }}_{sf}$). Some nanotubes reflected slip velocities (velocity jumps) at the interface. The slip velocity (*v*_*s*_) is a representation of the velocity of the slippage of the water core at the tube wall. Because of the slip velocity, water flow is greatly enhanced. However, the flow of the 12 nm-nanotubes with ${{\epsilon }}_{sf}=5$ or 10 kJ/mol is inhibited where it was obtained with no slip velocity (Fig. 1(b)). In Fig. 1, the no-slip Hagen–Poiseuille flow (which is a parabolic flow) is represented by a green-broken curve. A velocity profile located above the parabolic profile of the no-slip Hagen–Poiseuille flow indicates a flow enhancement. In contrast, a velocity profile is below the parabolic profile only if the flow is inhibited.

Whereas the plug-like flow of water in nanotubes was considered in previous studies^12,40–42^, we provide here evidence of two main flow inhibitors, which make water’s flow in nanotubes is neither parabolic nor plug-like. The first inhibitor is well known, which is the friction at the interface. This friction (inhibitor) defines the slippage of water over the tube wall. If the friction is higher than the hydrodynamics of water, the slip velocity is zero, and the flow is generally inhibited. It follows from Fig. 1 that the slip velocity decreases due to an increase in the water-surface interaction energy and/or nanotube’s radius. For water flow in 5 nm-nanotube, the slip velocity decreased by ~42% when ${{\epsilon }}_{sf}$ was increased from 0.1 to 10 kJ/mol (Fig. 1(a)). A reduction of ~44% was achieved when R was increased from 2.5 nm to 6 nm under ${{\epsilon }}_{sf}=0.1\,{\rm{kJ}}/{\rm{mol}}$ conditions. An increase in the friction force corresponds to an increase in the interfacial viscosity (Fig. 1). Generally, the friction force depends on the strength of the water-surface interaction. Thus, the magnitude of the friction force and the interfacial viscosity increase as ${{\epsilon }}_{sf}$ increases.

A second inhibitor is located at the first water layer. Beyond the interface, water was expected exhibiting a parabolic flow, and the corresponding viscosity of water was considered constant^8,12,16,25,26,42–44^. However, the flow of water is likely to be inhibited at the first water layer as shown in the velocity profiles in Fig. 1. The drag of water flow at this layer is due to a drag force that depends on water-surface interactions. Beyond the interface, the role of the interaction energy is switched to attract water particles resulting in a drag in the flow. An increase in the interaction energy is accompanied with an increase in the drag of the flow at the first water layer. As can be observed from Fig. 1, when ${{\epsilon }}_{sf}$ was increased from 0.1 to 10 kJ/mol, the drag increased by ~425% for 5 nm-nanotube and ~300% for 12 nm-nanotube. Moreover, the strength of the drag force is high just after the interface and decays as we go close to the nanotube center. This can be attributed to the fact that the interaction energy decays as the distance between water and the confining surface increases. When the drag force vanishes, the velocity profile is parabolic.

As one of the main findings of this study, it is revealed that the drag of water flow at the first water layer corresponds to an increase in the water viscosity (*μ*). The viscosity sharply increased at the first water layer, and then it decayed to the bulk water viscosity (*μ*_0_) near the nanotube center (Fig. 1). The viscosity increased to ~10 times the bulk water viscosity when ${{\epsilon }}_{sf}$ was increased from 0.1 to 10 kJ/mol. The increase in the viscosity at the first water layer is an expected result of the drag of the flow, which causes a reduction in the slope of water flow. According to the viscosity-slope relation (*i.e*. $\mu =-\,\frac{1}{2}pr/dv(r)/dr$), under a specific pressure gradient (*p*), the decrease in the slope (*dv*(*r*)/*dr*, *i.e*. *v*(*r*) is the velocity function) is accompanied with an increase in the viscosity (*μ*).

The existence of the drag of the water flow can be demonstrated from the literature. The drag of water flow can be observed in the velocity profiles obtained for the water shear by two polar hydrophilic surfaces^17^ and two mica surfaces^45^ where the velocity profiles were obtained curved over 0.4 nm distance from the confining surface. Moreover, the drag observed in Fig. 1 explains the “locking” at the boundary that was revealed in^46^ when water was sheared between two surfaces with strong water-surface interactions. For water in nanotubes, proofs of the existence of the drag and its localization at the first water layer are provided in Supplementary Information–S2 by presenting velocity profiles of water flow in polarized CNTs^47^. Here, we showed the interplay between the drag of water flow and its viscosity. We demonstrate via Fig. 1 that the drag of water flow at the first water layer is due to water-surface interactions, and it is associated with an increase in the water viscosity.

### Water Viscosity and Multiple Phases of Water

**Figure 2:**
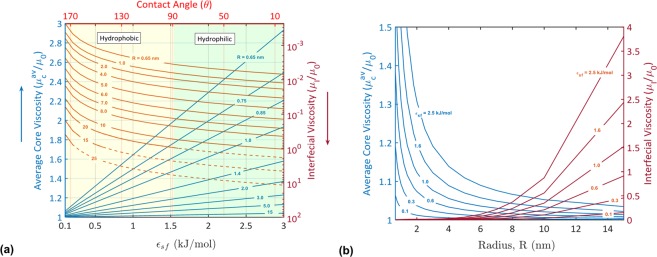
Effects of water-surface interactions and nanotube size on water viscosity. The interfacial viscosity (*μ*_*I*_) and average core viscosity (${\mu }_{c}^{av}$) as functions of (**a**) water-surface interactions (${{\epsilon }}_{sf}$)/contact angle (*θ*) and (**b**) the nanotube radius (*R*). Broken curves in (**a**) correspond to interfacial viscosities higher than bulk water viscosity. All the simulations were carried out with *σ*_*sf*_ = 0.3122 nm.

We demonstrate that the viscosity of water in nanotubes has a radial distribution. At the interface, water viscosity is constant, and it may decrease or increase depending on the relative hydrodynamics of the confined water and bulk water. Within the water core, the viscosity sharply increases due to water-surface interactions at the first water layer, and then it decays to the bulk water viscosity near the nanotube center. Thus, the drag observed in a velocity profile is conjugate to an increase in the water viscosity at the first water layer (Fig. 1). In Fig. 2, we report the influence of the water-surface interactions (${{\epsilon }}_{sf}$) and nanotube size (R) on the interfacial viscosity (*μ*_*I*_) and the average viscosity of the water core (${\mu }_{c}^{av}$) (The approach of determining the interfacial viscosity and core viscosity is explained in *Methods*). To investigate the interplay between the interfacial viscosity and the hydrophobicity/hydrophilicity of the confining surface (or surface wettability), the contact angle (*θ*) is related to the interaction energy (${{\epsilon }}_{sf}$) (${{\epsilon }}_{sf}-\theta$ relation is given in Supplementary Information - Fig. S4). The contact angle is the most accessible measure for the surface wettability. The contact angle changes from *θ* = 180° for a superhydrophobic surface to *θ* = 0° for a superhydrophilic surface. A decrease in the contact angle indicates a decrease in the surface hydrophobicity and a stronger water-surface interaction (Fig. S4). The interfacial viscosity and the average core viscosity are reported as functions of the contact angle (surface wettability) in Fig. 2. It should be mentioned that the contact angles presented in Fig. 2 and throughout this study refer to the wettability of the nanotube material. Thus, *θ* = 180° refers to a superhydrophobic surface near which a nanoconfined water flows very fast. *θ* = 0° refers to a superhydrophilic surface at which a nanoconfined water may slip over it^21,22^, stick to it^15^, or adsorbed through it^15^.

It follows from Fig. 2 that the interfacial viscosity (*μ*_*I*_) decreases due to a decrease in the water-surface interaction energy, a decrease in the confinement size, and/or an increase in the hydrophobicity of the nanotube. The confinement of water in a nanotube with a radius R < 10 nm causes a reduction in the interfacial viscosity lower than bulk water viscosity (*μ*_0_). For instance, the interfacial viscosity was intensively decreased to *μ*_*I*_ = 0.00023 mPa.s (viscosity of water vapor = 0.0097 to 0.0148 mPa.s^48^) when water was confined in a hydrophobic nanotube with R = 0.65 nm and ${{\epsilon }}_{sf}=0.1\,{\rm{kJ}}/{\rm{mol}}$. This value was increased to *μ*_*I*_ = 0.006 mPa.s when a hydrophilic nanotube was used with ${{\epsilon }}_{sf}=3\,{\rm{kJ}}/{\rm{mol}}$. This behavior can be attributed to the water depletion near the tube surface. The fraction of the water depletion in the interface region is high when water is confined in a hydrophobic nanotube. This fraction decreases as the hydrophilicity of the surface increases. Under severe confinement conditions, water may be depleted near a hydrophilic surface^15,20,21,31^, and hence the interfacial viscosity decreases lower than bulk water viscosity. When water is confined in large nanotubes (R > 10 nm), the interfacial viscosity may increase higher than bulk water viscosity depending on the surface wettability. The interfacial viscosity of hydrophilic nanotubes with R > 10 nm is greater than that of bulk water. For example, the interfacial viscosity of water in a nanotube with R = 25 nm is ~10 times that of bulk water. Generally, the viscosity of water interfaces with hydrophobic nanotubes is lower than that of water interfaces with hydrophilic nanotubes.

The viscosity at the first water layer increases higher than the one of bulk water due to an increase in the water-surface interaction energy and/or a decrease in the confinement size. This is because of the increase in the dag of water at the first water layer as ${{\epsilon }}_{sf}$ increases or R decreases. For example, the viscosity of water at this layer was obtained ~6 times that of bulk water when water was confined in a hydrophilic nanotube with R = 0.65 nm and ${{\epsilon }}_{sf}=3\,{\rm{kJ}}/{\rm{mol}}$. For hydrophobic nanotubes, the viscosity at the first water layer may increase up to ~3 times that of bulk water. Due to an increase in the viscosity at the first water layer, the average viscosity of the water core (${\mu }_{c}^{av}$) is generally increased higher than the bulk water viscosity (Fig. 2). The average core viscosity was obtained ~3 times that of bulk water when water was confined in a nanotube with R = 0.65 nm and ${{\epsilon }}_{sf}=3\,{\rm{kJ}}/{\rm{mol}}$. The obtained factors of increase of water viscosity within the core and at the first water layer are in agreement with water density profiles reported in various studies^5,12,15,23,24,44,49,50^. The density at the first water layer was obtained (1 to 4) times the density of bulk water.

Early investigations revealed the phase transitions of nanoconfined water into vapor and/or ice^5,6,30,40,51–54^. The severe confinement caused radial density variations, which were used to demonstrate the phase transitions and the multiphase structure of water in nanotubes. Here and for the first time, evidence of the formation of vapor and solid phases when water is intensively confined is provided based on the radial variation of water viscosity in the nanotube. Water viscosity changes are conjugate to its density changes due to a severe confinement^34,37,38^. Therefore, it is reliable to explore the phase transitions of water based on water viscosity.

**Figure 3:**
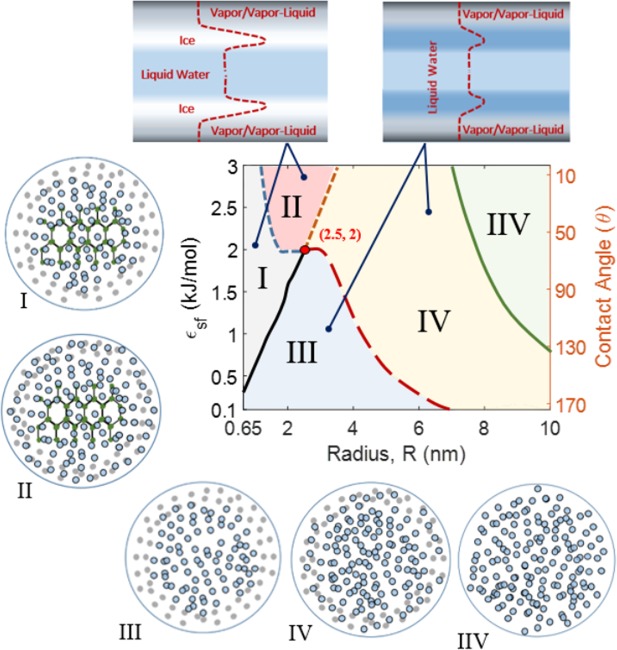
(${\epsilon }_{sf}/\theta -R$) phase diagram of confined water in hydrophobic and hydrophilic nanotubes at ambient conditions (300 K and 1 bar). Water in Regions I and II has a multiphase structure of vapor (Region I)/vapor-liquid mixture (Region II), ice, and liquid water. Water structure of Region III contains vapor at the interface and liquid water within the core. Within Region IV, vapor-liquid phase is formed at the interface and liquid water is located within the core. In Region IIV, a single-phase structure of liquid water is formed. The red bullet is a connection point (${{\epsilon }}_{sf}=2.5\,{\rm{kJ}}/{\rm{mol}}$, R = 2 nm) between four different phase structures of water. Schematics of the different structures of water within Regions I, II, III, IV, and IIV are given. On the top, illustrations are given for the viscosity distributions in nanotubes (dashed curves) and their corresponding different phases of water. All simulations were carried out with *σ*_*sf*_ = 0.3122 *nm*.

Using the viscosity data reported in Fig. 2, a diagram of the phase transitions of nanoconfined water in ${{\epsilon }}_{sf}-R$ plane and *θ* − *R* plane is developed (Fig. 3) (*see Methods*). The ${{\epsilon }}_{sf}/\theta -R$ phase diagram explores the different phases of water confined in hydrophobic and hydrophilic nanotubes of different sizes. When water is intensively nanoconfined (R ≤ 1.2 nm) and under weak water-surface interactions, the viscosity at the first water layer slightly increases, and a thick layer of vapor is formed at the interface (Region III). When confined in a hydrophilic nanotube (strong water-surface interactions), water exhibits a phase transition by the formation of a solid phase of water (ice) at the first water layer and a vapor phase at the interface (Region I). When water is confined in 1.2 nm < R < 2.5 nm nanotubes, water exhibits a different phase transition at high ${{\epsilon }}_{sf}$ values (hydrophilic nanotubes, is *θ* < 90°) by the formation of a multiphase structure of condensed vapor (vapor-liquid mixture) at the interface, ice at the first water layer, and liquid water in the core (Region II). The phase diagram shows a connection point (red bullet) that connects four different structures of water. At this point, water may exhibit any one of the structures of Regions I, II, III, and IV. With a slight left-shift from the connection point, the water phase transition due to an increase in ${{\epsilon }}_{sf}$ occurs by the formation of the solid phase followed by the formation of the vapor-liquid phase at the interface (Region I → Region II). On the other hand, to the slight right of the connection point, the phase transition with the increase in ${{\epsilon }}_{sf}$ occurs such that the vapor-liquid phase is formed first, and the ice phase formation comes after (Region IV → Region II). As the size of the confinement increases, water maintains its original single-phase structure of liquid water. Generally, the fraction of ice within the multiphase structure of water increases as ${{\epsilon }}_{sf}$ increases. In addition, the fraction of the vapor phase decreases due to a confinement size (R) increase, ${{\epsilon }}_{sf}$ increase, and/or an increase in the hydrophilicity of the confining surface (*θ* decreases lower than 90°).

Reported results in Figs 1–3 are in agreement with the existing findings regarding the viscosity of water interfaces with hydrophobic and hydrophilic flat surfaces^15,32,55–58^. At a hydrophilic surface, water depletion is very scarce and water particles may stick to the surface^32,55,57,58^ or even be absorbed through the surface^15^. Therefore, an experimental method can be proposed to determine the interfacial viscosity of water near hydrophilic flat surfaces (to the authors’ knowledge, all existing experiments were conducted for water on flat surfaces). For instance, using an interfacial force microscopy, Goertz *et al*.^55^ measured the interfacial viscosity of water on an amorphous silica surface. They revealed that the viscosity at a few nanometers separation from the hydrophilic surface is ~10^6^ times greater than that of bulk water. They attributed the increase in the interfacial viscosity to the hydrophilicity nature of the surface, which if degraded, such increase in viscosity would not occur. In another study, Li *et al*.^33^ used an atomic force microscopy method to determine the viscosity of water interfaces with different hydrophilic surfaces. They obtained viscosities with 4 orders-of-magnitude higher than bulk water viscosity. Major *et al*.^56^ measured the viscosity of water interface between oligoethyleneglycol-terminated alkanethiol self-assembled monolayer on an Au substrate by 7 orders-of-magnitude higher than that of bulk water. By shear resonance measurements, the viscosity of aqueous NaCl solution confined between two mica surfaces was determined of 2 to 4 orders-of-magnitude higher than the bulk water viscosity^57^. Using atomic force microscopy measurements, Ortiz-Young *et al*.^32^ obtained the interfacial viscosities of water with different hydrophilic surfaces. The viscosities were obtained 4 orders-of-magnitude higher than the bulk water viscosity. The calculations using our HCMM came in agreement with these observations where the viscosity of water interfaces at 0.35 nm separation from hydrophilic (*θ* < 90°) nanotubes with *R* = 1000 nm is obtained within the range (1.1 − 1.75) × 10^6^ times greater than the viscosity of bulk water.

In addition to experimental investigations, the obtained results in Figs 1–3 agree with the MD simulations, which were carried out to investigate the viscosity of water interfaces with hydrophilic flat surfaces. For example, the viscosity of a water film with <1 nm thickness confined between two mica surfaces was obtained 2 to 3 orders-of-magnitude higher than that of bulk water^45,58^. An ice-like layer was observed when water was confined between mica surfaces^28,29^ and silica surfaces^30^. Bonthuis and Netz^31^ carried out MD simulations of water interface between hydrophilic and hydrophobic flat surfaces. They calculated the interfacial viscosity of water with the hydrophilic surface 4 times that of bulk water while the interfacial viscosity with the hydrophobic surface is only 6.67% of the bulk water viscosity. Farimani and Aluru^34^ carried MD simulations to relate the viscosity of water confined in CNTs to its density. They demonstrated that an increase in the water density is associated with an increase in the water viscosity and a formation of an ice-like layer.

Because of the formation of a depletion layer, it is difficult to directly determine the viscosity of water interfaces with hydrophobic nanotubes using an experiment or a MD method. Therefore, most of the conducted studies on the viscosity of water in nanotubes assumed arbitrary values of the interfacial viscosity of water^16,25,26,31,34,43^. Here, we provide an effective approach to determine the interfacial viscosity as well as the radial distribution of water’s viscosity in hydrophilic and hydrophobic nanotubes (*see Methods*).

### Flow Enhancement/Inhibition

The flow of water in nanotubes would be enhanced or inhibited (Fig. 1). The flow rate is the most accessible measure that can be experimentally determined to quantify the enhancement/inhibition of water flow in nanotubes. Water flow is enhanced/inhibited by a factor (${\epsilon }$), which is the ratio of the flow rate of the nanoconfined water (*Q*_*C*_) to the no-slip Hagen–Poiseuille flow rate of bulk water (*Q*_*B*_). Different formulas were proposed to relate the flow rate of the confined water (*Q*_*C*_) to the slip-correction parameters (slip velocity (*v*_*s*_) and slip length (*L*_*s*_)) (*see* Supplementary Information-S1). Although the use of the *v*_*s*_ and *L*_*s*_ parameters is very common, we show in Supplementary Information-S1 that these parameters are insufficient and they should be replaced by a viscosity distribution-function. It was thought that Hagen–Poiseuille model reflects the same velocity profiles and flow rates as experimental and MD models when *v*_*s*_ and *L*_*s*_ parameters are involved. These parameters were derived assuming a parabolic flow after the initial velocity jump and a constant water viscosity. The drag in the water flow at the first water layer and the radial variation of the water viscosity revealed in Fig. 1 demonstrates the breakdown of *v*_*s*_ and *L*_*s*_ parameters. In view of these facts, here we provide a new formula that uses the radial variation of water viscosity to accurately calculate the enhancement/inhibition of water flow in nanotubes:

1 $${\epsilon }=\frac{2{\mu }_{0}}{{R}^{4}}(R{({\int }^{}\frac{r}{\mu (r)}dr)}_{r=R}-{\int }_{0}^{R}(r{\int }^{}\frac{r}{\mu (r)}dr)dr)$$

where *μ*_0_ is the viscosity of bulk water, and *μ*(*r*) is the radial variation of the water viscosity.

**Figure 4:**
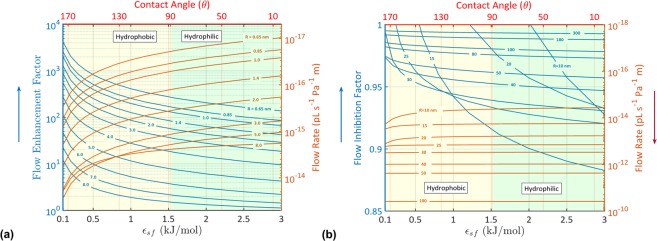
Effects of water-surface interactions and surface wettability on water’s flow rates in nanotubes. The flow enhancement factor (**a**) and inhibition factor (**b**) and their corresponding flow rates as functions of water-surface interactions and surface wettability. All simulations were carried out with *σ*_*sf*_ = 0.3122 *nm*.

In Fig. 4(a), we present cases of hydrophobic and hydrophilic nanotubes with enhanced water flows (${\epsilon } > 1$). The enhancement factor increases as the hydrophobicity of the confining surface increases (${{\epsilon }}_{sf}$ decreases and *θ* increases). For example, the enhancement factor of water in a nanotube with R = 0.65 nm increased from 135 to 4130 when ${{\epsilon }}_{sf}$ was decreased from 3 kJ/mol to 0.1 kJ/mol. This can be attributed to the increase in the flow rate due to an increase in the hydrophobicity (see Fig. 4(a)). An increase in the hydrophobicity decreases the interfacial viscosity and enhances the mechanism of forming water vapor at the interface. In contrast, the enhancement factor decreases due to an increase in the nanotube radius. This is because of the increase in the interfacial viscosity and the formation of vapor-liquid phase at the interface as the radius increases. Thus, the flow of water in large nanotubes approaches Hagen–Poiseuille flow.

With the existence of water-surface interactions, water flow in large nanotubes may be inhibited. In Fig. 4(b), we present cases of hydrophobic and hydrophilic nanotubes with inhibited flows (${\epsilon } < 1$). For example, the flow of water in a hydrophilic nanotube with R = 10 nm is slighted inhibited (${\epsilon }\, \sim \,0.93$). Reasons behind the flow inhibition are the increase in the viscosity at the first water layer (at high values of ${{\epsilon }}_{sf}$) and the existence of a liquid phase at the interface of these large nanotubes. It is worthy observing the decrease in the inhibition factor with the increase in the nanotube radius (Fig. 4(b)). Water maintains its original structure and conventional flow characteristics as the confinement size increases.

The obtained results in Fig. 4 are in agreement with the experimentally and computationally reported flow rates. Table 1 shows the flow rates and the flow enhancement\inhibition factors reported by experiments in parallel to the determined ones using the proposed HCMM. The results presented in Table 1 show the effectiveness of the proposed HCMM to simulate water flow in hydrophobic and hydrophilic nanotubes. In addition, the model was validated by comparison with over 90 cases of water flow in hydrophobic and hydrophilic nanotubes from the literature (*see Supplementary Information – Model Validation*). The results of our proposed HCMM are in agreement with data of MD in^12,25,28,59,60^ by <2% difference.

**Table 1:** Flow rates and flow enhancement/inhibition factors obtained by our proposed HCMM in comparison with experimental data available in the literature.

Nanotube	Radius (nm)	Flow rate per 1 (Pa/m) (m^3^/s) (10^−30^)		Enhancement/Inhibition		Ref.
		EXP	HCMM	EXP	HCMM	
CNT	21	1672	300	22	4	^8^
MWCNT	3.5	66–240	9.52–130	1120–4073	162–2206	^10^
DWCNT	0.65–1	0.28	0.114–0.3	713–4000	290–4280	^11^
Polycarbonate	7.5	4.6	2–2.2	3.7	1.61–1.77	^11^
CNT	0.405			882	793	^24^
CNT	0.435			662	487	^24^
CNT	0.49			354	255	^24^
CNT	0.71			103	75	^24^
CNT	0.76			59	60	^24^
CNT	0.795			51	54	^24^
BNNT	23			No slip	0.98	^67^
BNNT	26			No slip	0.95	^67^

## Discussion

The proposed HCMM is an effective approach to determine the radial distributions of viscosity of water confined in hydrophobic and hydrophilic nanotubes. Viscosity is determined depending on the water-surface interactions, wettability of the confining surface, and the confinement size (Eqs(9),(11), and (13) - *Methods*). When water is nanoconfined, residual interactions are generated between water particles and particles of the confining surface. These residual water-surface interactions influence the fluidity of water in nanotubes and cause phase transitions. In the absence of these residual interactions, water parabolically flows in tubes with a constant viscosity. However, because of the water-surface interactions, water flow in nanotubes is non-parabolic, and the viscosity has a radial distribution (Fig. 1).

Here, evidence were given to demonstrate that water flow in nanotubes is neither a parabolic flow nor a plug-like flow. Water flows in nanotubes under the influence of two inhibitors. An inhibitor in the form of a friction force influences the slippage of water particles over the confining nanotubular surface. If the hydrodynamics of water outweighs the action of the friction force, water flow is enhanced and flow profiles with velocity jumps at the interface are obtained (Fig. 1). The second inhibitor is located at the first water layer in the form of a drag force. Because of this drag force, water flow is greatly inhibited at the first water layer (Fig. 1). Under the action of these two inhibitors, water flow is non-parabolic everywhere inside the nanotube (except within the region very close to the tube center, the flow may be parabolic). Because of the revealed non-parabolic nature of water flow in nanotubes, the hydrodynamic boundary conditions (slip boundary conditions) defined by Navier, which were proposed based on a slip length (*L*_*s*_ = *μ*/*η*) and a slip velocity (${v}_{s}={L}_{s}\times |{(dv(r)/dr)}_{r=R}|$) breakdown (*see Supplementary Information*–S1). These boundary conditions were proposed to compensate water flow for the friction at the interface (*η* is the coefficient of friction) assuming a constant viscosity (*μ*). Here, we showed the effectiveness of replacing the hydrodynamic boundary conditions by a radial distribution of viscosity to secrete accurate descriptions of water flow in nanotubes. In addition, an accurate description of the enhancement/inhibition of flow of water in hydrophobic and hydrophilic nanotubes was provided in Eq.(1) and Fig. 4.

It is of interest to highlight the possibility of scaling water flow rate using a radial distribution of water viscosity. A previous study proposed a scaling parameter of water flow enhancement based on the diffusion coefficient^61^. Here, we showed that water flow is generally enhanced when *μ*_*I*_ < *μ*_0_, and it is inhibited when *μ*_*I*_ > *μ*_0_ (see Figs 2 and 4). The flow rate can be scaled to the slope of the water flow at the interface, which is inversely proportional to the *μ*_*I*_/*μ*_0_ ratio. In addition to *μ*_*I*_/*μ*_0_ ratio, the flow rate depends on water viscosity at the first water layer. As the viscosity of water at the first water layer increases, the flow rate decreases. Thus, based on the observations derived in this study, a unique scaling parameter that depends on water viscosity can be proposed.

The proposed HCMM effectively interprets the phase transitions of confined water in hydrophobic and hydrophilic nanotubes (Fig. 3). The knowledge of the phase behavior of confined water is crucial to understand the so many nontraditional phenomena of nanofluidics^6^. The transition to a multiphase structure is expected when water is nanoconfined. At the interface and under the ambient conditions, in the case of a weak water-surface interaction, a transition to the vapor phase is expected (Region III) while a transition to a vapor-liquid mixture phase is expected under a strong water-surface interaction (Region II). These findings totally agree with the early observations of the dry transition and wetting transition of water near substrates with interactions with water^6^. It is worthy observing the formation of a pure vapor phase at interfaces of, both, hydrophobic and hydrophilic nanotubes when water is severely confined (R ≤ 1.2 nm). Under a strong water-surface interaction, a solid phase of water confined in a nanotube with R ≤ 3.5 nm is expected at the first water layer (Regions I and II). This solid phase is formed prior to/after the formation of a vapor-liquid phase at the interface. Whereas the transitions of bulk water confined in nanotubes into vapor and/or ice have been discussed in previous studies^34,49,53,54,62,63^, the sequence of the formation of the vapor and ice phases when water confinement conditions are changed is not clear. Here, we provide information about the evolution of the phase transition of water, which are important when design nanofluidics. In addition, at the connection point (red bullet in Fig. 3), whereas the same flow rate is obtained, water is expected to exhibit one of four different structures (Regions I, II, III, and IV). Thus, for the complete description of nanoconfined water, both the fluidity and phase transitions are needed.

Whereas water phase transitions into vapor/ice would be important from the fundamental point of view, information about the specific confinement conditions required for the design of such systems has not been demonstrated for the practical use. The experiment by Agrawal *et al*.^54^ showed extreme diameter-dependent phase transitions of water into ice/vapor when confined in CNTs over a range of diameters from 1.05 to 1.52 nm. Here, we provide in Fig. 3 the diameter and wettability-dependent phase transitions of nanoconfined water over a wide range of diameters from 0.65 nm to 10 nm and a range of water-surface interaction potentials from ${{\epsilon }}_{sf}=0.1\,{\rm{kJ}}/{\rm{mol}}$ to ${{\epsilon }}_{sf}=3\,{\rm{kJ}}/{\rm{mol}}$. We believe that these findings are important for the design of advanced water-based nanofluidics systems in the future.

Finally, it should be mentioned that the reported viscosity data in this study are derived by considering water is a continuum system. However, the use of these data when the water system is converted into a sub-continuum system should be done with care. Thus, still the transition-boundary from continuum to sub-continuum flow of water needs to be defined before using the viscosity information provided here. To define this boundary, an approach that depends on MD simulations, like the one presented in^26^, would be used.

In summary, we have put the fluidity and phase transition of confined water in hydrophobic and hydrophilic nanotubes under scrutiny by interpreting the interplay between the flow and viscosity of water. We reported the distribution of water viscosity in nanotubes as a function of the water-surface interaction energy, surface wettability, and nanotube size. In addition, a phase diagram that explores water phase transitions depending on the water-surface interaction energy, surface wettability, and nanotube size was provided. We revealed a water drag at the first water layer, which is conjugate to an increase in the water viscosity and a formation of a solid water phase under a severe confinement (R ≤ 3.5 nm) and a strong water-surface interaction. We demonstrated the transition of water phase into a vapor/vapor-liquid phase at the interface. We reported a point at which the solid phase may be formed before/after the formation of a vapor-liquid phase at the interface. In view of the revealed fact that water may exhibit different structures under the same flow rate, measurements of the permeability of nanoporous membranes^10,64^ and designs of nanofluidics^3^, water desalination systems^65^, and fog collectors^66^ should be carried out with reasoning investigations of fluidity and phase transitions of the confined water. Indeed, the findings of this study provide answers to some of the existing questions regarding the role of the surface wettability^5^ and water-surface interactions^6^ to the phase transitions of confined water.

## Methods

Simulations presented in this study were carried out using a new hybrid continuum-molecular mechanics (HCMM). This new mechanics depends on an effective approach for identifying the viscosity of water flow in hydrophobic and hydrophilic nanotubes. Thus, new measures were developed and incorporated to correct Hagen–Poiseuille model for water-surface interactions and surface wettability.

### Hagen–Poiseuille Model Modified for Water-Surface Interactions

A water particle flows under the influence of a set of interaction forces generated between the particle and the other surrounding water particles. When the water particle approaches a solid surface, residual interaction forces are generated between the particle and all the particles forming the solid surface (*see* Fig. S10–Supplementary Information). The strength of these residual interactions depends on the distance between the water particle and the solid surface. The residual water-solid interactions are usually neglected when water is macroscopically confined. However, when water is nanoconfined, the contribution of these residual interactions is considerable and affects the water flow. A detailed derivation of the model presented here is given in Supplementary Information – S6.

Accounting for the nearest residual water-surface interactions, the equilibrium equation of the steady state flow of water with a viscosity *μ*_0_ under a pressure gradient within a nanotube with a radius *R* is derived as follows:

2 $$\begin{array}{lll}-p-\frac{1}{r}\frac{d}{dr}(r(\tau (r)+t(r)))=0 & i.e. & 0\le r\le R\end{array}$$

where *τ*(*r*) is the viscous stress conjugate to the water-water interactions. *t*(*r*) is introduced as a residual viscous stress that accounts for water-surface interactions. These viscous stress measures are related to the velocity of water particles as follows:

3 $$\tau (r)=-\,{\mu }_{0}\frac{dv(r)}{dr}\,$$

4 $$t(r)=-\,{\mu }_{sf}(r)\frac{dv(r)}{dr}$$

where *μ*_0_ is the conventional viscosity of bulk water which is conjugate to water-water interactions. Here, we introduce *μ*_*sf*_ (*r*) as a new viscosity measure due to water-surface interactions. It should be noted that *μ*_0_ is constant while *μ*_*sf*_ (*r*) varies in the radial direction. *μ*_*sf*_ (*r*) depends on the separation between a water particle at *r* and the nanotube surface (the separation is *R* − *r*).

By introducing *T*(*r*) = *τ*(*r*) + *t*(*r*) as the total viscous stress:

5 $$T(r)=-\,\mu (r)\frac{dv(r)}{dr}\,$$

The effective viscosity of water in nanotubes accounting for the water-surface interactions can be defined as follows:

6 $$\begin{array}{lll}\mu (r)={\mu }_{0}(1+\xi (r)) & {\rm{with}} & \xi (r)=\frac{{\mu }_{sf}(r)}{{\mu }_{0}}\end{array}$$

Eq. (6) presents a correction of bulk water viscosity for water-surface interactions.

Challenges of the utilization of the slip boundary conditions at water-solid interfaces are discussed in (Supplementary Information–S1). It is demonstrated that the description of water flow using the slip boundary conditions is insufficient to describe accurately the nontraditional phenomena of nanoconfined water, and these slip boundary conditions should be replaced by a radial distribution of the water viscosity.

Because the radial distribution of water viscosity is considered, the slip boundary conditions can be effectively skipped (*see* Supplementary Information – S1) and the following no-slip boundary conditions can be applied:

7 $$T(0)=0\,{\rm{and}}\,v(R)=0$$

Consequently, the velocity profile of water flow through a nanotube is derived as follows:

8 $$v(r)=\frac{p}{2}[{({\int }^{}\frac{r}{\mu (r)}dr)}_{r=R}-{\int }^{}\frac{r}{\mu (r)}dr]$$

where *p* denotes the pressure gradient.

### Water Viscosity in Nanotubes (*μ*(*r*))

To determine the radial distribution of water viscosity, the structure of water in the nanotube is considered. Because of the water-surface interactions and the surface wettability, water interface with the nanotube wall is a totally different phase^7,34,40^. Therefore, water viscosity profile shows a sharp decrease/increase at the interfacial layer (Figs S1 and 1). In the water core, the viscosity sharply increases at the first water layer and then decays to the bulk water viscosity (Figs S1 and 1). As a result, water can be modeled as a water core with a radial viscosity distribution, *μ*_*c*_(*r*), and an interfacial-shell with a viscosity, *μ*_*I*_. Thus, the viscosity of water in nanotubes can be defined as follows:

9 $$\mu (r)=\{\begin{array}{ll}{\mu }_{I} & (R-\delta )\le r\le R\\ {\mu }_{c}(r)={\mu }_{0}(1+\xi (r))\, & r < (R-\delta )\end{array}$$

where *δ* is the interface thickness which is considered by *δ* = 1.1224${s}_{sf}$^7^. This thickness, *δ*, equals the water–surface separation at which the water–surface attraction force is zero.

### Determination of Interfacial Viscosity (*μ*_*I*_)

Here, an approach is proposed to determine the viscosity of water interfaces with hydrophobic and hydrophilic nanotubes as functions of the nanotube size and water-surface interaction energy. First, over 20 cases of MD simulations of water flow in CNTs from the literature were used to derive the slip velocity-to-pressure gradient ratio (VPR) as a function of the CNT radius (see Fig. S3). With the aid of the VPR function, the interfacial viscosity as a function of the CNT radius can be determined as follow^7^:

10 $${\mu }_{I}(R,\theta \cong 155^\circ )=\frac{{R}^{2}-{(R-\delta )}^{2}}{VPR(R)}$$

where $\theta \cong 155^\circ$ is the average contact angle of CNTs^16^.

The interfacial viscosity depends on the surface wettability^16,17,27,43^. For a superhydrophobic surface, the contact angle *θ* = 180° and ${{\epsilon }}_{sf}\to 0$. For a superhydrophilic surface, *θ* = 0°. The contact angle can be linearly related to the water-surface interaction energy^16,17,27,43^. Therefore, with the linear interpolation between *θ* = 180° and ${{\epsilon }}_{sf}\to 0$ for a superhydrophobic surface and *θ* ≅ 155° and ${{\epsilon }}_{sf}=0.4247\,{\rm{kJ}}/{\rm{mo}}l$ for CNTs (these are the average values of *θ* and ${{\epsilon }}_{sf}$ considered in literature), the following relation can be derived:

11 $${\mu }_{I}(R,{{\epsilon }}_{sf})=2.355{{\epsilon }}_{sf}(\frac{{R}^{2}-{(R-\delta )}^{2}}{VPR(R)})$$

where VPR is plotted as a function of the nanotube radius in Fig. S3. Figure S4 shows the contact angle as a function of the water-surface interaction energy (${{\epsilon }}_{sf}$).

### Determination of Water Core Viscosity (*μ*_*c*_(*r*))

The viscosity of water core sharply increases at the first water layer due to water-surface interactions. In fact, the rise in the water viscosity at this layer depends on the relative hydrodynamics of the confined water and bulk water. Because the viscosity of water and its hydrodynamics depend on the interatomic potential, *ξ*(*r*) (introduced in Eq. (9)) is defined as the ratio of the water-water interatomic force to the water-surface interatomic force. By modeling water-surface interactions based on *LJ* potential, *ξ*(*r*) is derived in the form:

12 $$\xi (r)=-\,\frac{5{{\epsilon }}_{sf}{\sigma }_{ff}}{3{{\epsilon }}_{ff}{\sigma }_{sf}}[12{(\frac{{\sigma }_{sf}}{R-r})}^{13}-6{(\frac{{\sigma }_{sf}}{R-r})}^{7}]$$

Thus, the viscosity of water core is obtained in the form:

13 $${\mu }_{c}(r)={\mu }_{0}(1-\frac{5{{\epsilon }}_{sf}{\sigma }_{ff}}{3{{\epsilon }}_{ff}{\sigma }_{sf}}[12{(\frac{{\sigma }_{sf}}{R-r})}^{13}-6{(\frac{{\sigma }_{sf}}{R-r})}^{7}])$$

### Phase Diagram

The phase diagram presented in Fig. 3 is developed based on the interfacial and the average core viscosities reported in Fig. 2. The data of the performed simulations were used to determine the phase transition lines (shown in Fig. 3). Different phases of water were considered: solid phase (ice), liquid water, vapor, and vapor-liquid mixture. The transitions between these different phases were defined based on the interfacial viscosity and the average core viscosity, according to Table 2:

**Table 2:** The interfacial viscosity and the average core viscosity for the transitions between vapor, vapor-liquid mixture, liquid, and ice phases of water.

Phase	Viscosity (mPa.s)	Location in Nanotube
Vapor	0.0097 ≤ *μ*_*I*_ ≤ 0.0148^48^	Interface
Vapor-Liquid Mixture	0.0148 < *μ*_*I*_ ≤ 0.276^68^	Interface
Liquid Water	0.276 < *μ*_*I*_ ≤ 1.8^68^	Interface
Ice	${\mu }_{c}^{av}\ge 1.2\,$	First water layer

## Supplementary information

Supplementary Information

## Data Availability

The data that support the findings of this study are included in the article and Supplementary Information.
