# Supplementary material for: Fluidity and phase transitions of water in hydrophobic and hydrophilic nanotubes
Source: Sci Rep. 2019 Apr 5;9:5689. doi: 10.1038/s41598-019-42101-4 (PMC6450949; doi:10.1038/s41598-019-42101-4)
Supplement: Supplementary file 1 — Supplementary Information [file 41598_2019_42101_MOESM1_ESM.pdf]

Supplementary Information

Fluidity and phase transitions of water in hydrophobic and hydrophilic nanotubes

Mohamed Shaat[1,2,3] and Yongmei Zheng[4]

[1] Department of Mechanical Engineering, Zagazig University, Zagazig 44511, Egypt.

[2] Mechanical Engineering Department, Abu Dhabi University, Abu Dhabi, P.O.BOX 59911, United Arab Emirates.

[3] Engineering and Manufacturing Technologies Department, DACC, New Mexico State University, Las Cruces, NM 88003, USA.

[4] Key Laboratory of Bio-Inspired Smart Interfacial Science and Technology of Ministry of Education School of Chemistry, and Beijing Advanced Innovation Center for Biomedical Engineering Beihang University (BUAA) Beijing 100191, P. R. China.

E-mail: shaatscience@yahoo.com; shaat@nmsu.edu (M. Shaat)
zhengym@buaa.edu.cn (Y. Zheng)

Contents

S1: Slip Boundary Conditions Should be replaced by a Radial Distribution of Viscosity

S2: Drag of Water Flow at the First Water Layer

S3: VPR Function

S4: Relation between Water-Surface Interaction Energy and Surface Wettability

S5: Model Validation

S6: Modified Navier-Stokes Equation

References

Correspondence and requests for materials should be addressed to M. Shaat (email: shaat@nmsu.edu ; shaatscience@yahoo.com).

S1: Slip Boundary Conditions Should be replaced by a Radial Distribution of Viscosity

Nanoconfined water slips over a hydrophobic surface[1], and it may stick to or leak through a hydrophilic surface[2]. Therefore, the conventional no-slip boundary conditions at the water-surface interfaces do not hold. Over the past years, various experimental and molecular dynamics (MD) models were proposed to report the slip boundary conditions of nanoconfined water[3]. Utilizing these slip boundary conditions at different water-surface interfaces, the classical continuum models of fluid mechanics were thought they can reflect the same results as experimental and MD models. However, many challenges are associated with this approach. Here, we discuss some of these challenges.

The slip boundary conditions of water in nanotubes were defined as follows[3,4]:

$$v(R) = v_s \text{ or } v(R + L_s) = 0 \qquad \text{(S1)}$$

where $v(r)$ is the velocity profile function. v_s and L_s are slip correction parameters. v_s is the slip velocity represents the velocity jump at the interface. L_s is the slip length, which is the linear/nonlinear extrapolation of the velocity profile to a radius at which velocity would be zero. The slip length was related to the slip velocity via a linear extrapolation as follows[3,4]:

$$L_s = \frac{v_s}{\left|\left(\frac{dv(r)}{dr}\right)_{r=R}\right|} \qquad \text{(S2)}$$

Here, we demonstrate that the slip parameters (L_s and v_s) of the boundary conditions are insufficient to describe accurately the nontraditional phenomena of nanoconfined water, and these parameters should be replaced by a radial distribution of the water viscosity.

Depending on the nanotube's size and wettability, the slip parameters attain values of $v_s \geq 0$ and $\pm L_s \geq 0$. A non-zero slip velocity, $v_s > 0$, is an indication of water slippage over the nanotube wall, and a zero slip velocity gives the conventional Hagen–Poiseuille model. Thus, the slip velocity is not a proper parameter to model the drag revealed in Fig.1 (see the main text) or the absorption[2] of water particles at the boundary with hydrophilic surfaces. The slip length, however, could be positive or negative. For water slippage over the surface, the slip length is

positive. A negative slip length indicates a sticking of water particles to the surface and a drag in the water flow. Thus, the slip length is more efficient than the slip velocity to quantify the enhancement/inhibition of water flow in nanotubes. Therefore, different formulas were proposed to relate the flow enhancement/inhibition factor (ϵ) to the slip length (L_s). These formulas are collected in Table S1.

Table S1: Formulas of the enhancement/inhibition factor-slip length relation ($\epsilon - L_s$) reported in the literature.

$(\epsilon - L_s)$ **Relation**	**Assumptions**	**Ref.**
$\epsilon = 1 + \frac{4L_s}{R}$	- L_s is linearly extrapolated (Eq. (S2)).<br>- Constant viscosity of water in nanotube.<br>- The profile of water flow has a velocity jump at the interface followed by a parabolic flow.	4
$\epsilon = \left(1 + \frac{4L_s(R)}{R}\right)\frac{\mu_0}{\mu(R)}$<br>where<br>$L_s(R) = 30\text{ nm} + \frac{C}{(2R)^3}$<br>$C$ is a fitting parameter.	- L_s is linearly extrapolated (Eq. (S2)).<br>- The profile of water flow has a velocity jump at the interface followed by a parabolic flow.<br>- Constant water viscosity within the nanotube. However, an effective water viscosity that depends on the nanotube radius was considered.<br>- The effective viscosity is the weighted average of an arbitrary assumed interfacial viscosity (~0.655 mPa·s) to the bulk water viscosity.	5
$L_s = \left(\frac{\mu_0}{k}\right)\left(\frac{v_s}{v_0 \operatorname{arcsinh}\left(\frac{v_s}{v_0}\right)}\right)$<br>$k$ is the dynamic coefficient of friction. v_0 (m/s) is a fitting parameter.	- L_s is linearly extrapolated (Eq. (S2)).<br>- Constant viscosity of water in nanotube.<br>- The profile of water flow has a velocity jump at the interface followed by a parabolic flow.<br>- $L_s \geq 0$.	6
$\epsilon = 1 + \frac{4L_s(R)}{R}$<br>where<br>$L_s(R) = \delta\left(\frac{\mu_I}{\mu_c} - 1\right)\left[1 - \frac{3}{2}\frac{\delta}{R} + \left(\frac{\delta}{R}\right)^2 - \frac{1}{4}\left(\frac{\delta}{R}\right)^3\right]$	- L_s is linearly extrapolated (Eq. (S2)).<br>- Constant viscosity of water in nanotube.<br>- The profile of water flow has a velocity jump at the interface followed by a parabolic flow.<br>- The slip length depends on the nanotube size.<br>- Distinguish between water viscosity at the interface (μ_I) and viscosity of water core (μ_c).	7
$\epsilon = \left(1 + \frac{4L_s(\theta)}{R}\right)\frac{\mu_0}{\mu(R)}$<br>where<br>$L_s(\theta) = \frac{C}{(\cos\theta + 1)^2}$<br>$C$ is a fitting parameter.<br>θ surface contact angle.	- L_s is linearly extrapolated (Eq. (S2)).<br>- The profile of water flow has a velocity jump at the interface followed by a parabolic flow.<br>- Constant water viscosity within the nanotube. However, an effective water viscosity that depends on the nanotube radius was considered.<br>- The effective viscosity is the weighted average of the interfacial viscosity to the bulk water viscosity.<br>- The interfacial viscosity (μ_I) is related to the surface wettability: $\mu_I = \mu_0(-0.018\theta + 3.25)$<br>- The slip length depends on the surface wettability.	8

There is a growing debate about the true value of the slip length of water flow in nanopores[3,9]. Discrepancies between the reported values of the slip length in the literature can be observed and have been discussed in previous studies[3,9]. Moreover, big discrepancies between slip lengths of experimental studies and slip lengths obtained from MD were discussed[3,9]. Here, we demonstrate that the $\epsilon - L_s$ relations in Table S1 are insufficient and this is the reason behind these discrepancies. These relations are only limited for a special case of water flow in nanotubes. These relations were derived assuming that the velocity jump is followed by a parabolic velocity profile within the water core and a constant distribution of water viscosity within the nanotube. However, because of the water drag at the first water layer (see Fig.1 in the main text), these relations cannot properly reflect the enhancement/inhibition of water flow in hydrophobic/hydrophilic nanotubes.

For further demonstration, we present in Fig. S1 two cases of water flow. In previous investigations, water flow was assumed like case A (Fig.S1(a)). In this case, water particles were considered sliding over the wall (thick black line) where a jump in the velocity profile (blue) was considered at the water interface with the tube. The velocity profile, $v(r)$, within the water core was assumed parabolic. According to these assumptions, the slope of the velocity (brown) linearly decreases within the water core because of the parabolic flow, and the slope of the velocity at the interface sharply decreases due to water slippage. According to the viscosity-slope relation (i.e. $\mu(r) = -\frac{1}{2}pr/dv(r)/dr$ where p is the pressure gradient), the viscosity of the water core is constant (green), and it sharply decreases at the interface. For Case A, the nonlinear extrapolation is more accurate than the linear extrapolation (Fig.S1(a)). None of the $\epsilon - L_s$ relations in Table S1 can accurately model water flows like Case A. All relations were derived based on $\epsilon = 1 + \frac{4L_s}{R}$, which assumes a linear extrapolation of the slip length. However, the accuracy of these relations can be modified via a nonlinear extrapolation of the slip length, and, in this case, these relations can give an accurate representation of water flow of Case A. However,

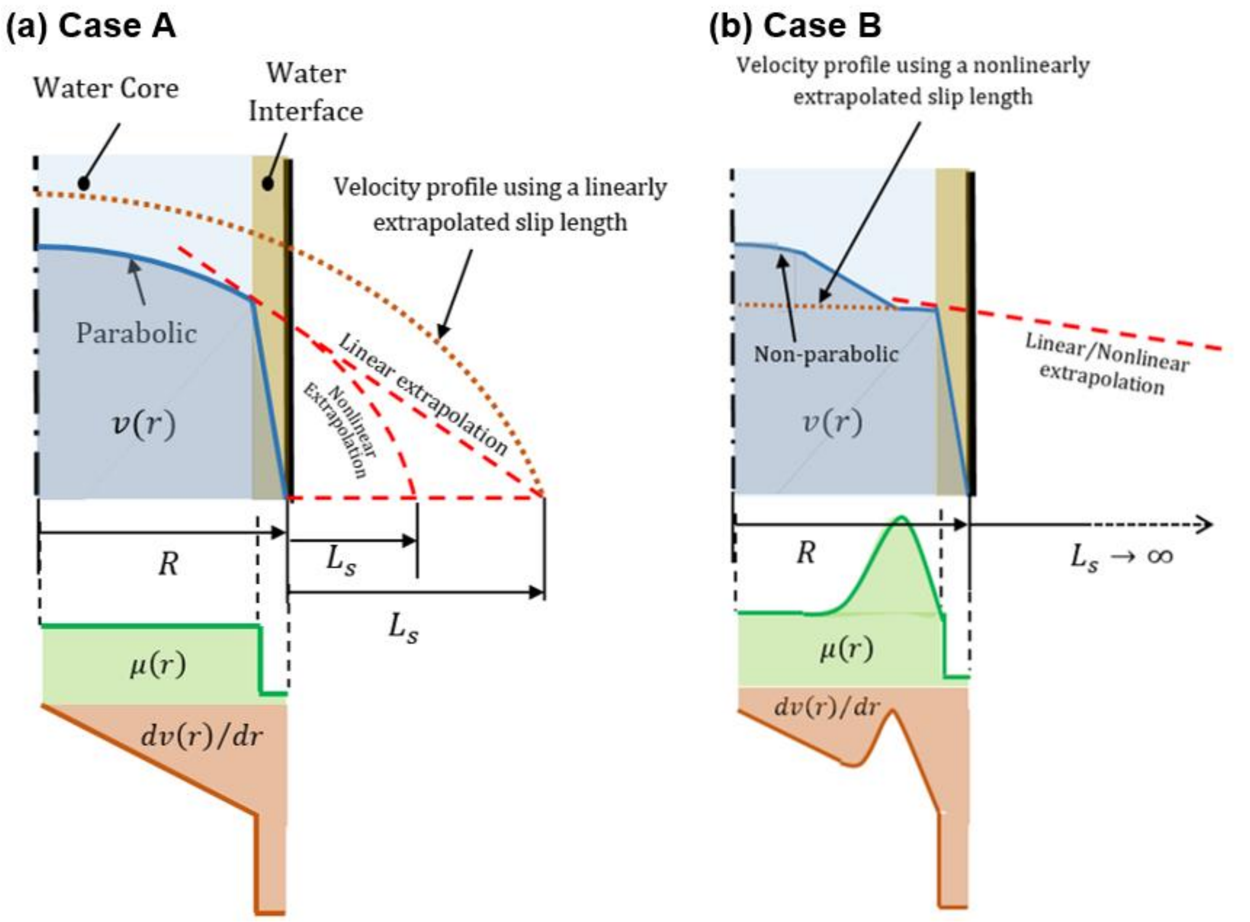

Figure S1: Evaluation of the slip length as a measure of enhancement/inhibition of water flow in nanotubes. (a) Case A: water flow in nanotubes neglecting effects of water-surface interactions on water particles at the first water layer. The flow is parabolic within the core with a velocity jump at the interface. For this case, the slope of the velocity is as shown by the brown schematic, and the viscosity is constant (green). A nonlinearly extrapolated slip length gives an accurate representation of this virtual case of water flow. (b) Case B: an actual water flow in nanotubes considering the influence of water-surface interactions on water particles at the first water layer. The flow is generally non-parabolic, and the viscosity has a radial distribution, as shown. The slip length cannot extrapolate this case. For an accurate representation of the actual water flow (Case B), a radial distribution function of water viscosity should replace the slip boundary conditions.

because of the water-surface interactions, the actual water flow in nanotubes is similar to Case B (Fig.S1(b)). Non-parabolic velocity profiles with a jump at the interface are usually observed when water flow in nanotubes (see Fig.1). The linear and (even) nonlinear extrapolations give wrong

corrections of Hagen–Poiseuille flow, as shown in Case B. The viscosity, however, is a proper measure that can effectively reflect the changes in the velocity profiles due to water-tube interactions and the surface wettability. This non-parabolic velocity profile indicates that the viscosity of water radially varies as presented in Fig.S1(b).

S2: Drag of Water Flow at the First Water Layer

Majumder and Corry[10] carried out MD simulations of water flow in polarized CNTs to investigate effects of the electrostatic interactions between polar water particles and polar CNT walls. They added charges to the CNT wall to increase the interaction between water and CNT. Because of the additional electrostatic potential, water-CNT interactions were increased from $\epsilon_{sf} = 1.423\ \mathrm{kJ/mol}$ (for nonpolarized CNT) to $\epsilon_{sf} = 6.95\ \mathrm{kJ/mol}$ (for polarized CNT with +/- 0.5 e charge) and $\epsilon_{sf} = 18\ \mathrm{kJ/mol}$ (for polarized CNT with +/- 0.9 e charge). The flow of water in a nonpolarized CNT was obtained with a negligible drag (Fig.S2). The increase in the water-CNT interaction, however, resulted in a drag in the water flow at the first water layer. The increase in the water-CNT interaction increases the hydrophilicity in the system where water flow approaches to the parabolic flow of bulk water. In addition to the water-surface interactions, roughness of the confining surface causes a drag at the first water layer (see the case of Rough CNT in Fig. S2).

It should be mentioned that Majumber and Corry[10] did not refer to the drag of water flow at the first water layer. Instead, they demonstrated that, due to high electrostatic interactions or high roughness between water and the confining surface, the enhancement of water flow was affected and the flow converted to be a parabolic flow. Here, we show that the increase in the water-surface interaction and/or the roughness of the confining surface are accompanied with a drag at the first water layer, which is associated with a sharp increase in water viscosity at this layer.

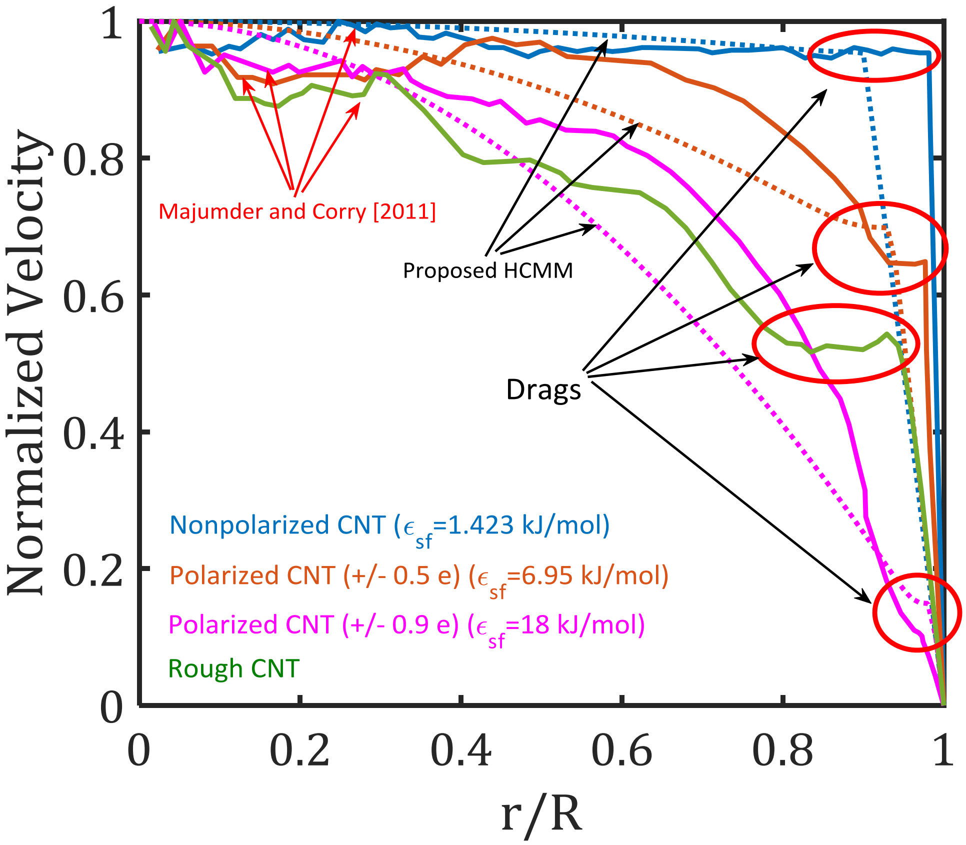

Figure S2: Proof of drag of water flow due to water-surface interactions. Drags of water flow are red-circled. Results of the proposed hybrid continuum-molecular mechanics (HCMM) for water flow in nonpolarized and polarized CNTs are plotted for comparison to results of MD simulations performed by Majumder and Corry[10]. The proposed HCMM effectively gives the drag of water flow at the first water layer. For comparison, velocity profiles are normalized with respect to the corresponding maximum velocity.

S3: VPR Function

The velocity-to-pressure gradient ratio (VPR) is plotted as a function of the nanotube radius based on the results of MD simulations of water flow in CNTs[5,9,11–16]. It should be mentioned that the first author previously used this approach to identify the interfacial viscosity of water flow in CNTs[17].

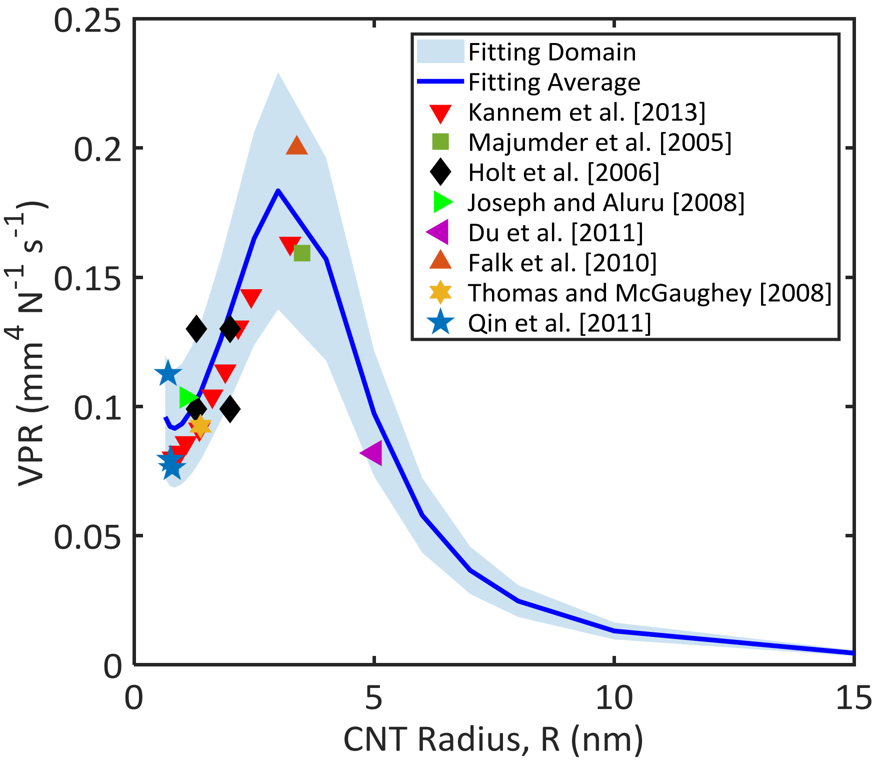

Figure S3: Determination of the VPR function.

S4: Relation between Water-Surface Interaction Energy and Surface Wettability

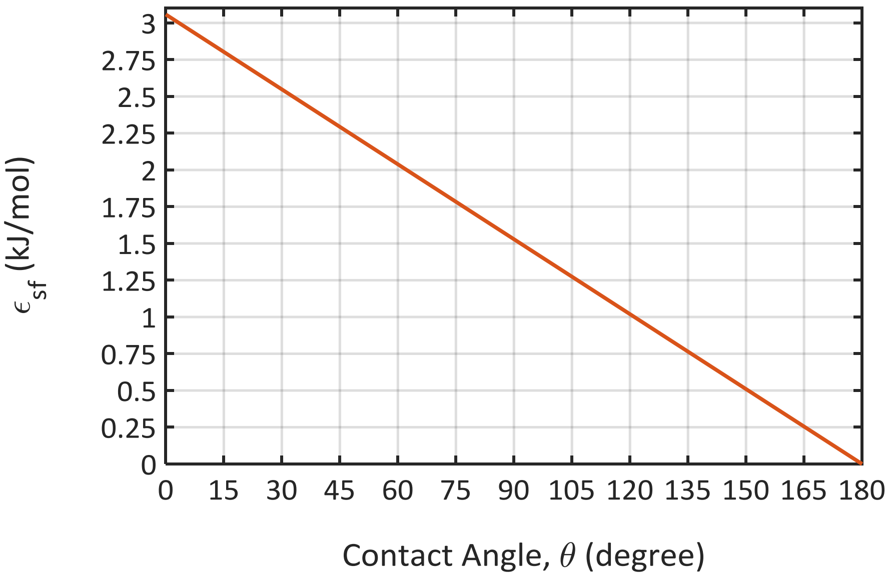

Figure S4: Relation between water-surface interaction energy (ϵ_{sf}) and contact angle (θ) as a measure of the surface wettability.

S5: Model Validation

To validate the proposed hybrid continuum-molecular mechanics (HCMM), tests were carried out by comparing the results obtained by the proposed HCMM to the results of experimental and MD models available in the literature.

Comparison to Whitby et al.[4] Using a nanoporous membrane with 43 ± 3 nm CNTs, Whitby et al.[4] reported the flow characteristics of water through the membrane. The pressure was measured for different imposed flow rates through the membrane. The density of the CNTs in the membrane was determined by 1.07×10^{10} $\#/\mathrm{cm}^2$. Table S2 shows the flow rates as determined by the proposed HCMM in comparison to the experimentally determined ones.

Table S2: Comparison to Whitby et al.[4] (Membrane thickness is $76\ \mu\mathrm{m}$ and diameter of the CNT is 42 nm).

**Imposed Flow Rate ($\mathbf{m^3/s}$)**	**Measured Pressure (kPa)**	**Flow Rate per a CNT (EXP) ($\mathbf{m^3/s}$)**	**ϵ (EXP)**	**Flow Rate per a CNT (HCMM) ($\mathbf{m^3/s}$)**	**ϵ (HCMM)**
4.167×10^{-10}	1.38 - 1.94	3.89×10^{-20}	20 - 24	$(3.76 \rightarrow 5.29) \times 10^{-21}$	2.713
8.34×10^{-10}	2.91 - 3.16	7.79×10^{-20}	20 - 24	$(7.9 \rightarrow 8.62) \times 10^{-21}$	2.713
1.25×10^{-9}	4.21 - 5.425	1.168×10^{-19}	20 - 24	$(1.15 \rightarrow 1.48) \times 10^{-20}$	2.713
1.67×10^{-9}	6.078 - 7.04	1.56×10^{-19}	20 - 24	$(1.66 \rightarrow 1.92) \times 10^{-20}$	2.713
2.12×10^{-9}	7.7 - 9	1.98×10^{-19}	20 - 24	$(2.1 \rightarrow 2.45) \times 10^{-20}$	2.713

Comparison to Majumder et al.[13] The experimental setup in Majumder et al.[13] measures the permeability of a membrane of multi-walled carbon nanotube (MWCNTs). The inner core diameter of nanotubes was 7 nm and the density of pores was determined by 5×10^{10} per cm^2. Using the proposed HCMM, simulations are run for a nanotube with a contact angle of $176°$ [8]. The flow rate and the flow velocity are calculated and compared to the experimentally determined ones as follows:

Table S3: Comparison to Majumder et al.[13].

**Pore Density (# per $\mathbf{cm^2}$)**	**Membrane Thickness (μm)**	**Initial Permeability (cm^3/cm^2-min-bar)**	**Average Velocity at 1 bar (cm/s)**		**Flow Rate ($\mathbf{cm^3/s}$) ($\mathbf{10^{-13}}$)**	
			**EXP**	**HCMM**	**EXP**	**HCMM**
5×10^{10}	34 - 126	0.58 – 1.01	0.5 – 0.875	0.08 – 0.29	1.934–3.367	(0.28–1.035)

Comparison to Holt et al.[11] Holt et al.[11] reported water flow measurements through membranes with aligned double-walled carbon nanotubes DWCNTs serve as pores with diameters of less than 2 nm. They measured the permeability of three membranes with 20, 3, and 2.8 μm thicknesses and compared it to the permeability of polycarbonate membrane. The density of pores was estimated by $\leq 0.25 \times 10^{12}$ $\#/cm^2$ for the DWCNTs membranes and 6×10^8 $\#/cm^2$ for polycarbonate membrane. Table S4 shows the measured permeability of the membranes in comparison to the calculated one using the proposed HCMM.

Table S4: Comparison to Holt et al.[11].

Membrane	Pore Diameter (nm)	Thickness (μm)	Measured Permeability ($m^3/s - cm^2 - atm$)	Flow Rate per Pore ($m^3/s - atm$)		Flow Enhancement	
				EXP	HCMM	EXP	HCMM
DWNTs #1	1.3 – 2	2	7.5×10^{-9}	3.0×10^{-20}	5.77×10^{-21} - 1.56×10^{-20}	1500-8400	783 – 1626
DWNTs #2	1.3 – 2	3	2.33×10^{-9}	9.32×10^{-21}	3.85×10^{-21} - 1.04×10^{-20}	680-3800	783 – 1626
DWNTs #3	1.3 – 2	2.8	1.92×10^{-9}	7.68×10^{-21}	4.12×10^{-21} - 1.11×10^{-20}	560-3100	783 – 1626
Polycarbonate	15	6	0.047×10^{-9}	7.8×10^{-20}	3.43×10^{-20} - 3.64×10^{-20}	3.7	1.64 - 1.735

Comparison to Qin et al.[14] Qin et al.[14] proposed an approach for measuring the enhancement of water flow on ultra-long single-CNTs using a field effect transistors array[14]. In the carried out experiment by Qin et al.[14], water flowed under the influence of an electrical field where the flow velocity was determined by measuring the change in the electrical current. They used a CNT with a total length $1330\ \mu m$ where the flow of water is damped over a damping length of $280\ \mu m$. To compensate the damping effect due to the electrical field, an equivalent length of the CNT of 0.025 m is used in the performed analyses. Qin et al.[14] reported the velocity of water flow on CNTs and the enhancement in the flow velocity (ratio of the measured velocity to the Hagen–Poiseuille velocity). Fig. S5 shows their results in comparison to the results of the proposed HCMM.

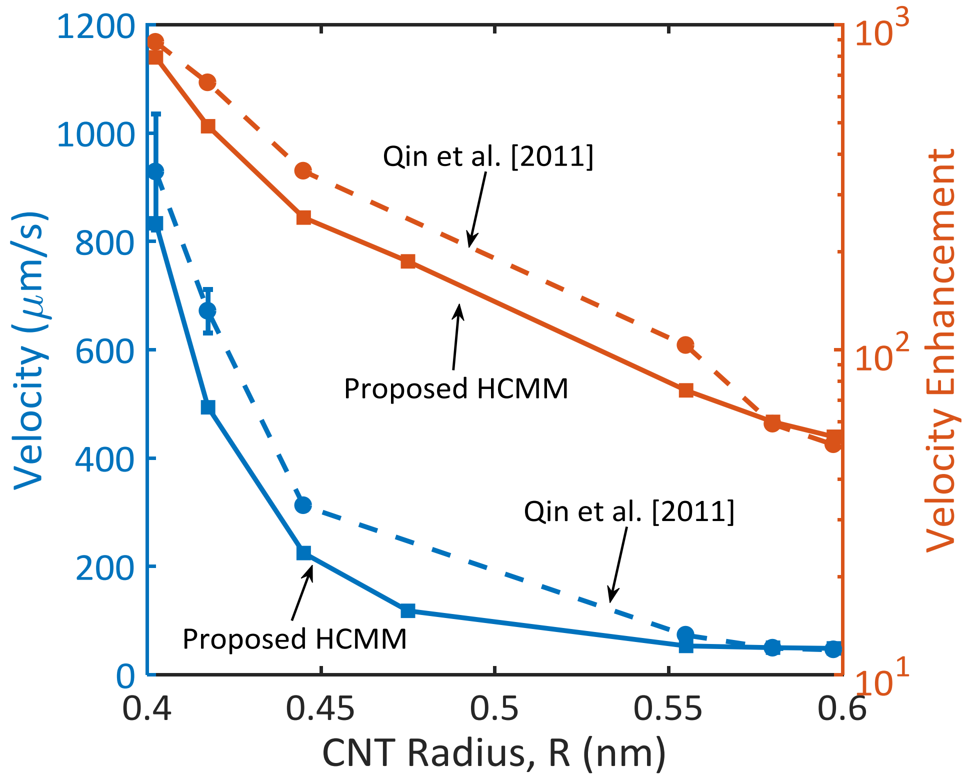

Figure S5: Comparison to Qin et al.[14]**.** Flow velocity and velocity enhancement as functions of the CNT radius.

Comparison to Kannam et al.[9] Kannam et al.[9] carried out MD simulations of water flow in CNTs with different sizes. They reported the slip velocity, slip length, and enhancement for water flow in CNTs with different sizes. Figs. S6, S7, and S8 show comparisons between the results of the proposed HCMM and results of MD simulations presented in[9].

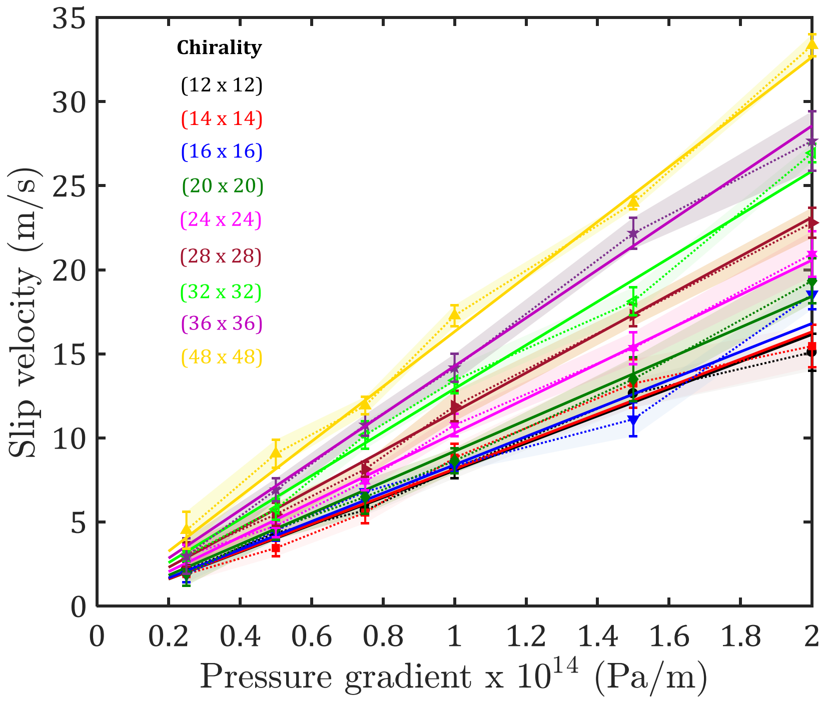

Figure S6: Slip velocity as a function of the pressure gradient for water flow in CNTs. Symbols with error bars and shaded regions refer to the results of the MD simulations[9]. Solid lines are the results of the proposed HCMM.

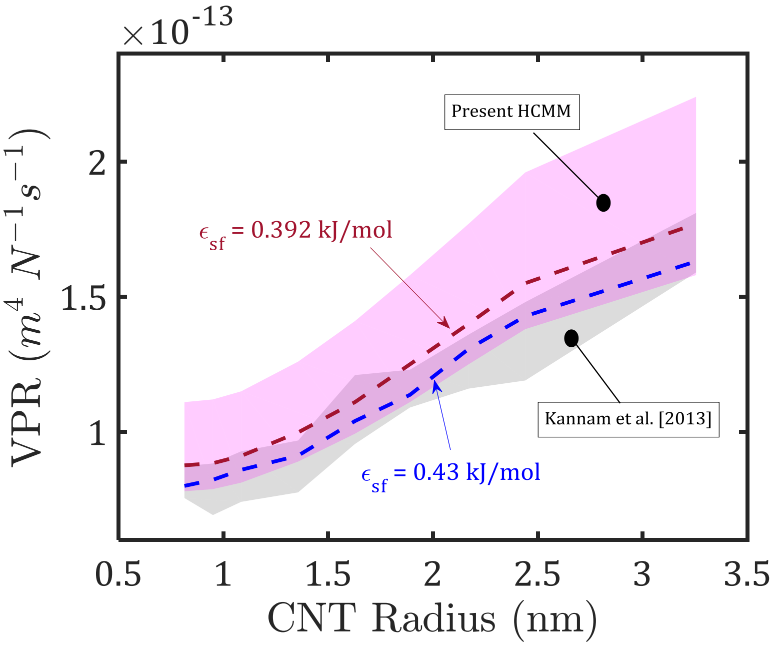

Figure S7: Velocity to Pressure gradient Ratio (VPR) as a function of the CNT radius. The results of the proposed HCMM (pink) are compared to the results of MD simulations[9] (gray). The dashed lines represent the results of the proposed HCMM for two different values of ϵ_{sf}.

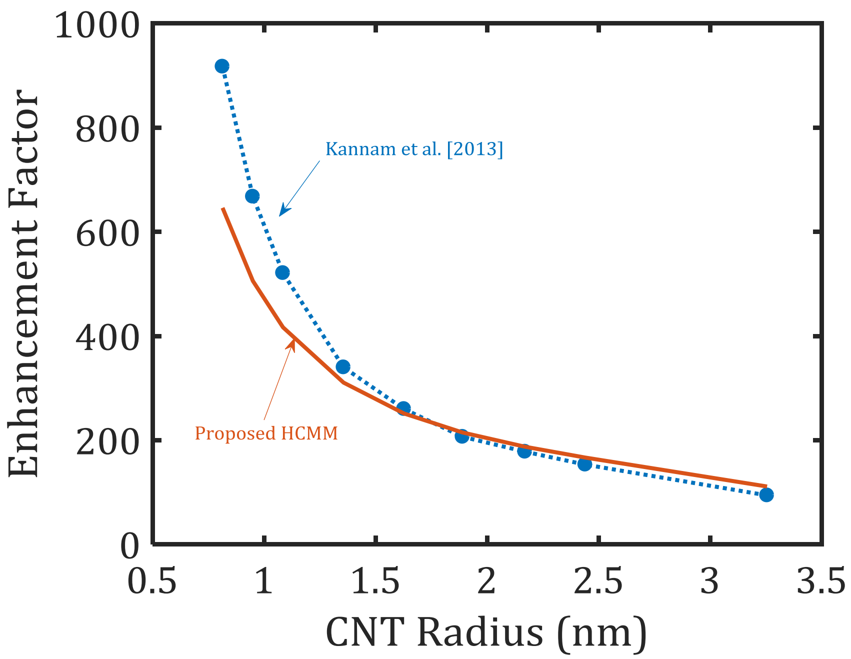

Figure S8: Flow enhancement as a function of the CNT radius. The results of the proposed HCMM (solid curve) are compared to the results of MD[9] (doted curve).

Comparison to Milischuk and Ladanyi[18] and Chiavazzo et al.[19] for Silica pores Milischuk and Ladanyi[18] carried out MD simulations of water flow in silica nanopores with 2 to 4 nm diameters. They reported a decrease in the diffusivity with a decrease in the nanopore diameter, which indicated an enhancement in the flow of water due to water confinement[18,19]. The energy parameters used in the MD simulations carried out by Milischuk and Ladanyi[18] were $\varepsilon_{sf} = 1.912\ \mathrm{kJ/mol}$ and $\sigma_{sf} = 0.27\ \mathrm{nm}$. Table S5 shows the results of the performed analyses using the proposed HCMM.

Table S5: Enhancement factor (ϵ) of water flow in silica nanotubes (obtained using the proposed HCMM).

Nanopore	Diameter (nm)	Core viscosity mPa.s	Enhancement Factor (ϵ)
Silica	2	1.56	96.79
Silica	3	1.32	55.74
Silica	4	1.23	40.744
Silica	8.13	1.103	12.36
Silica	11.04	1.0744	3.84

The proposed HCMM revealed that the flow of water is enhanced as the nanotube diameter decreases. This matches the observations on the diffusivity decrease presented by Milischuk and Ladanyi[18] and Chiavazzo et al.[19]. Wu et al.[8], however, showed that the water flow in Silica nanopores is inhibited and instead a negative slip length was obtained (the slip length was determined by -0.3 to -0.22). The results of Wu et al.[8] contradict with the observations of the original studies.

Comparison to Joseph and Aluru[12] and Thomas and McGaughey[5]

Figure S9 shows a comparison between velocity profiles obtained by the proposed HCMM and the ones of MD simulations[5,12] for water flow in a CNT, a BNNT, and a nanotube with LJ parameters were defined for Si (NT with Si LJ).

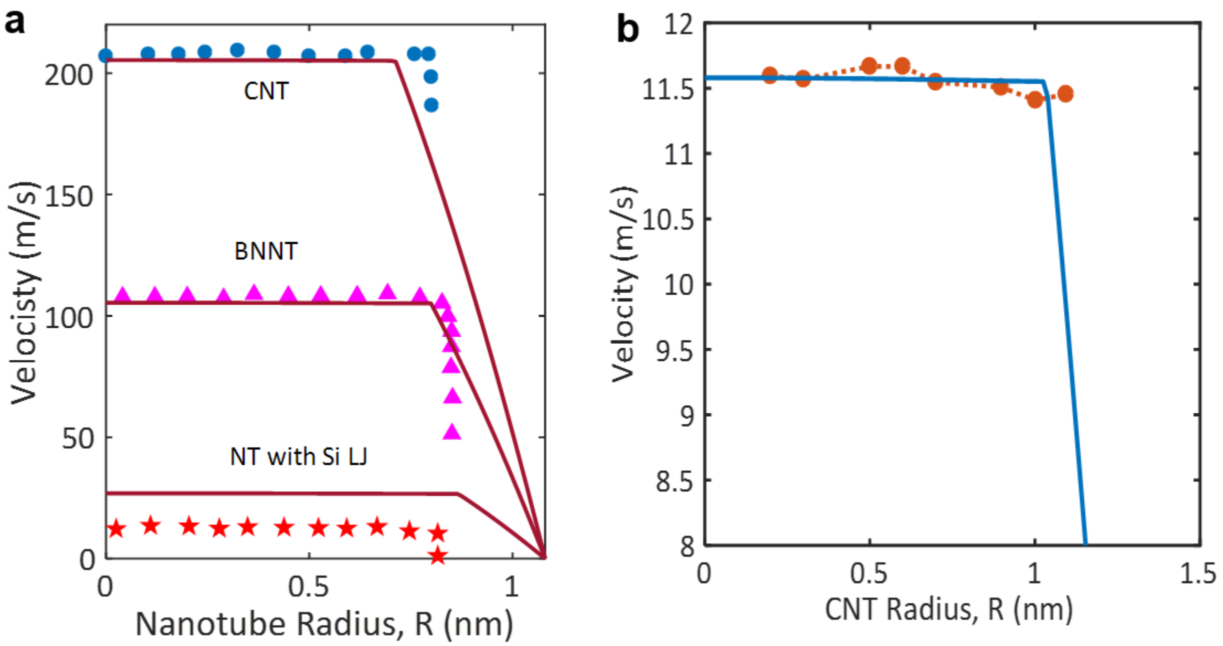

Figure S9: (a) Comparison to Joseph and Aluru et al.[12]. Velocity profiles of water flow in a CNT, a BNNT, and a nanotube with LJ parameters were defined for Si (NT with Si LJ) (pressure gradient, $p = 2 \pm 0.4 \times 10^{15}\ \mathrm{Pa/m}$ and $R = 1.08\ \mathrm{nm}$). **(b)** Comparison to Thomas and McGaughey[5]. Velocity profile of water flow in a CNT ($p = 1.24 \times 10^{14}\ \mathrm{Pa/m}$ and $R = 1.385\ \mathrm{nm}$). Results of the proposed HCMM are presented by solid curves.

S6: Modified Navier-Stokes Equation

Here, a detailed formulation of the model presented in *Methods* is given. Consider a water particle at a position $\boldsymbol{x}$ in the space. This water particle is surrounded by a set of other water particles and is located close to a solid surface, as shown in Fig.S10. Interactions between this water particle and other water particles are generated. The strength of each one of these interactions is $\boldsymbol{F}_{ff}$ (this interaction force is conjugate to the water-water interactions). In addition, to $\boldsymbol{F}_{ff}$, a residual interaction force is generated between the water particle and the nearest particle of the solid surface ($\boldsymbol{F}_{sf}$), as shown in Fig.S10. The strength of the residual interaction depends on the distance between the water particle and the solid particle. Now, Newtonian's balance conditions can be applied to the water particle to give:

$$\begin{aligned} &\sum \boldsymbol{F}_{ff} + \boldsymbol{F}_{sf} = m\dot{\boldsymbol{v}} \\ &\sum \left(\mathbf{X} \times \boldsymbol{F}_{ff}\right) + \left(\mathbf{X} \times \boldsymbol{F}_{sf}\right) = \mathbf{X} \times m\dot{\boldsymbol{v}} \end{aligned} \qquad \text{(S3)}$$

where $m\dot{\boldsymbol{v}}$ is the momentum of the water particle, which is balanced by the water-water interactions and water-solid interactions.

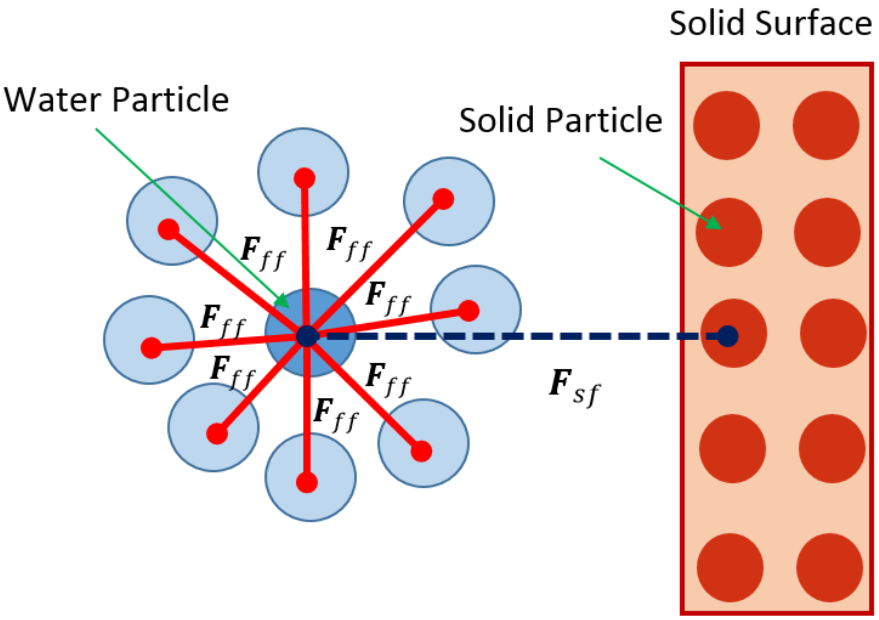

Figure S10: A schematic of a water particle under water-water interactions and water-surface interactions.

For a nanoconfined water occupies a volume Ω and bounded by a surface S, according to Eq.(S3), the continuity, balance of momentum, balance of moment of momentum equations can be, respectively, obtained as follows:

$$\frac{\partial}{\partial t}\left(\int_{\Omega} \rho \, d\Omega\right) = -\int_{\Omega} (\boldsymbol{\nabla} \cdot \rho \boldsymbol{v}) \, d\Omega \tag{S4}$$

$$\int_{\Omega} \boldsymbol{F} \, d\Omega + \int_{S} \boldsymbol{T}^{(n)} \, dS = \int_{\Omega} \rho \dot{\boldsymbol{v}} \, d\Omega \tag{S5}$$

$$\int_{\Omega} (\mathbf{X} \times \boldsymbol{F}) \, d\Omega + \int_{S} \left(\mathbf{X} \times \boldsymbol{T}^{(n)}\right) dS = \int_{\Omega} (\mathbf{X} \times \rho \dot{\boldsymbol{v}}) \, d\Omega \tag{S6}$$

where ρ is the mass density. $\boldsymbol{F}$ denotes a body force vector. $\boldsymbol{T}^{(n)} dS$ denotes the water's surface tractions. It should be mentioned that $\boldsymbol{T}^{(n)}$ is the sum of the convective momentum fluxes and all the molecular momentum fluxes. Because of the water-solid interaction, the molecular momentum flux tensor is formed depending on two viscous momentum flux tensors, as follows:

$$\boldsymbol{T}^{(n)} = -(\boldsymbol{n} \cdot \boldsymbol{\phi})$$
with (S7)
$$\boldsymbol{\phi} = P\boldsymbol{I} + \boldsymbol{\tau} + \boldsymbol{t} + \rho \boldsymbol{v}\boldsymbol{v}$$

where $\boldsymbol{n}$ is the unit normal vector and $\boldsymbol{I}$ is the unit tensor. $\boldsymbol{\phi}$ denotes the combined momentum-flux tensor. P is the applied pressure field.

In Eq.(S7), $\boldsymbol{\tau}$ denotes the viscous stress tensor conjugate to the water-water interactions while $\boldsymbol{t}$ is the viscous stress tensor that is conjugate to the water-solid interactions. It should be noted that the momentum-flux tensor and the viscous stress tensor presented in Eqs.(S7) are general tensors, which can be decomposed into symmetric and skew-symmetric parts.

According to Eq.(S7) and the divergence theorem, the continuity and balance equations of a water continuum can be written as follows:

$$\frac{\partial}{\partial t}\rho = -\boldsymbol{\nabla} \cdot \rho \boldsymbol{v} \tag{S8}$$

$$\frac{\partial}{\partial t}(\rho \boldsymbol{v}) = -\boldsymbol{\nabla} \cdot \rho \boldsymbol{v}\boldsymbol{v} - \boldsymbol{\nabla} P - \boldsymbol{\nabla} \cdot \boldsymbol{\tau} - \boldsymbol{\nabla} \cdot \boldsymbol{t} + \rho \boldsymbol{g} \tag{S9}$$

$$(\boldsymbol{\tau} + \boldsymbol{t}) \times \boldsymbol{I} = 0 \tag{S10}$$

where the gravitational force, $\rho \boldsymbol{g}$, is introduced as a body force.

Equation (S10) indicates that the skew-symmetric parts of the viscous stress tensors, $\boldsymbol{\tau}$ and $\boldsymbol{t}$**,** vanish. Thus, the balance equations (Eq.(S9)) can be rewritten as follows:

$$\frac{\partial}{\partial t}(\rho \boldsymbol{v}) = -\boldsymbol{\nabla} \cdot \rho \boldsymbol{v}\boldsymbol{v} - \boldsymbol{\nabla} P - \boldsymbol{\nabla} \cdot \boldsymbol{\tau}^{sym} - \boldsymbol{\nabla} \cdot \boldsymbol{t}^{sym} + \rho \boldsymbol{g} \tag{S11}$$

where the balance equations depend on the symmetric part of the viscous stress tensors, $\boldsymbol{\tau}^{sym}$ and $\boldsymbol{t}^{sym}$.

It should be mentioned that Eq.(S11) represents a modified Navier-Stockes equation for the water-surface interactions. Thus, if water-surface interactions are neglected, Eq.(S11) reduces to the conventional Navier-Stockes equation.

The viscous stress tensors can be formed based on Newton's law of viscosity as follows:

$$\boldsymbol{\tau}^{sym} = -\mu_0(\boldsymbol{\nabla}\boldsymbol{v} + \boldsymbol{v}\boldsymbol{\nabla}) \tag{S12}$$

$$\boldsymbol{t}^{sym} = -\mu_{sf}(\boldsymbol{x})(\boldsymbol{\nabla}\boldsymbol{v} + \boldsymbol{v}\boldsymbol{\nabla}) \tag{S13}$$

where μ_0 denotes the viscosity of bulk water. μ_{sf} is a newly introduced viscosity to account for water-solid interactions.

To reset Eq.(S11) to the conventional Navier-Stockes equation, the two stresses $\boldsymbol{\tau}$ and $\boldsymbol{t}$ are summed into an equivalent total viscous stress $\boldsymbol{T} = \boldsymbol{\tau} + \boldsymbol{t}$. Then, this stress can be formed based on Newton's law of viscosity:

$$\boldsymbol{T} = -\left(\mu_0 + \mu_{sf}(\boldsymbol{x})\right)(\boldsymbol{\nabla}\boldsymbol{v} + \boldsymbol{v}\boldsymbol{\nabla}) = -\mu(\boldsymbol{x})(\boldsymbol{\nabla}\boldsymbol{v} + \boldsymbol{v}\boldsymbol{\nabla}) \tag{S14}$$

where

$$\mu(\boldsymbol{x}) = \mu_0 + \mu_{sf}(\boldsymbol{x}) = \mu_0\left(1 + \xi(\boldsymbol{x})\right) \tag{S15}$$

where $\xi(\boldsymbol{x}) = \mu_{sf}(\boldsymbol{x})/\mu_0$.

References

1. Sendner, C., Horinek, D., Bocquet, L. & Netz, R. R. Interfacial water at hydrophobic and hydrophilic surfaces: Slip, viscosity, and diffusion. *Langmuir* **25,** 10768–10781 (2009).
2. Janeček, J. & Netz, R. R. Interfacial water at hydrophobic and hydrophilic surfaces: Depletion versus adsorption. *Langmuir* **23,** 8417–8429 (2007).
3. Kannam, S. K., Daivis, P. J. & Todd, B. D. Modeling slip and flow enhancement of water in carbon nanotubes. *MRS Bull.* **42,** 283–288 (2017).
4. Whitby, M., Cagnon, L., Thanou, M. & Quirke, N. Enhanced fluid flow through nanoscale carbon pipes. *Nano Lett.* **8,** 2632–2637 (2008).
5. Thomas, J. A. & Mcgaughey, A. J. H. Reassessing Fast Water Transport Through Carbon Nanotubes. *Nano Lett.* **8,** 2788–2793 (2008).
6. Ma, M. D. *et al.* Friction of water slipping in carbon nanotubes. *Phys. Rev. E - Stat. Nonlinear, Soft Matter Phys.* **83,** 1–7 (2011).
7. Myers, T. G. Why are slip lengths so large in carbon nanotubes? *Microfluid. Nanofluidics* **10,** 1141–1145 (2011).
8. Wu, K. *et al.* Wettability effect on nanoconfined water flow. *PNAS* **114,** 3358–3363 (2017).
9. Kannam, S. K., Todd, B. D., Hansen, J. S. & Daivis, P. J. How fast does water flow in carbon nanotubes? *J. Chem. Phys.* **138,** 094701 (2013).
10. Majumder, M. & Corry, B. Anomalous decline of water transport in covalently modified carbon nanotube membranes. *Chem. Commun.* **47,** 7683–7685 (2011).
11. Holt, J. K. *et al.* Fast mass transport through sub – 2-nanometer carbon nanotubes. *Science (80-.).* **312,** 1034–1037 (2006).
12. Joseph, S. & Aluru, N. R. Why Are Carbon Nanotubes Fast Transporters of Water? *Nano Lett.* **8,** 452–458 (2008).
13. Majumder, M., Chopra, N., Andrews, R. & Hinds, B. J. Nanoscale hydrodynamics: Enhanced flow in carbon nanotubes. *Nature* **438,** 44 (2005).
14. Qin, X., Yuan, Q., Zhao, Y., Xie, S. & Liu, Z. Measurement of the Rate of Water Translocation through Carbon Nanotubes. *Nano Lett.* **11,** 2173–2177 (2011).
15. Falk, K., Sedlmeier, F., Joly, L., Netz, R. R. & Bocquet, L. Molecular origin of fast water transport in carbon nanotube membranes: Superlubricity versus curvature dependent friction. *Nano Lett.* **10,** 4067–4073 (2010).
16. Du, F., Qu, L., Xia, Z., Feng, L. & Dai, L. Membranes of vertically aligned superlong carbon nanotubes. *Langmuir* **27,** 8437–8443 (2011).
17. Shaat, M. Viscosity of Water Interfaces with Hydrophobic Nanopores: Application to Water Flow in Carbon Nanotubes. *Langmuir* **33,** 12814–12819 (2017).
18. Milischuk, A. A. & Ladanyi, B. M. Structure and dynamics of water confined in silica nanopores. *J. Chem. Phys.* **135,** 174709 (2011).
19. Chiavazzo, E., Fasano, M., Asinari, P. & Decuzzi, P. Scaling behaviour for the water transport in nanoconfined geometries. *Nat. Commun.* **5,** 1–11 (2014).
